# Augmented reality to visualize nerve segmentation intraoperatively for endoscopic discectomy

**DOI:** 10.3171/2026.4.FOCVID2650

**Published:** 2026-07-01

**Authors:** Isabella E. Decker, Felix Toussaint, Meriem Boukhiam, Jordan Hatfield, Muhammad M. Abd-El-Barr

**Affiliations:** Department of Neurosurgery, Duke Health, Durham, North Carolina

**Keywords:** endoscopic surgery, endoscopic transforaminal discectomy, augmented reality, nerve segmentation

## Abstract

Intraoperative visualization throughout endoscopic discectomy remains limited. A 70-year-old female presented with a left-sided L2–3 disc herniation. Preoperative MRI-based nerve segmentation was integrated with intraoperative CT imaging to generate a virtual operative field. Augmented reality (AR) visualization was used to project the segmented spinal anatomy onto the patient in real time. A partial discectomy was performed to decompress the affected nerves without the need for a foraminoplasty. The patient tolerated the procedure well with significant pain relief. This case study demonstrates the safety and feasibility of endoscopic discectomy without foraminoplasty with the aid of AR guidance with preoperative nerve segmentation.

The video can be found here: https://stream.cadmore.media/r10.3171/2026.4.FOCVID2650

## Transcript

### 0:24 Background.

Endoscopic lumbar discectomy through Kambin’s triangle offers the promise of focused decompression with preservation of native stabilizing structures, yet the corridor remains unforgiving. This right-angled space between the exiting nerve root, the superior articular process, and the superior vertebral body endplate is narrow, highly variable, and further restricted at the lumbar levels, in scoliosis, and in facet hypertrophy.^1^ Most transforaminal endoscopic techniques navigate this space through fluoroscopic bony landmarks supplemented by intraoperative electromyography or intraoperative feedback as a functional safeguard. This approach advances the trajectory toward the disc without any direct 3D representation of the individual course of the exiting nerve root. The decision to proceed without foraminoplasty therefore reflects the surgeon’s confidence rather than precise knowledge of the true nerve-free working corridor. Recent neuroimaging advances have begun to reshape this landscape. Advanced MRI and nerve segmentation map segmental roots relative to the disc and have been proposed as preoperative planning tools in transforaminal surgery.^2^ Specifically, studies applying nerve segmentation to Kambin’s triangle show increased variability in size and position of nerve-free regions across levels and across patients.^3^ In parallel, segmentation workflows have matured to convert routine magnetic resonance and CT data into explicit 3D models of the exiting and traversing roots, the thecal sac, the disc, and the adjacent vertebrae. Augmented reality extends these advantages into the operative field. In fact, early reports show that segmented neural overlays derived from 3D MRI can be aligned to intraoperative imaging.^4^ This renders nerve position a continuous visual landmark during transfacet and transforaminal fusion, rather than an inferred construct.^5^ Image-guided endoscopic discectomy has also been described with 3D neuronavigation that improves docking accuracy.^6,7^ Nevertheless, published experience combining a fully segmented 3D nerve model, fused to CT, with intraoperative visualization throughout endoscopic transforaminal discectomy in the setting of severe deformity remains limited. We report a case in which such an approach was applied in a patient with chronic lumbar scoliosis and debilitating radiculopathy.

### 2:38 Clinical Presentation.

A 70-year-old female with chronic lumbar scoliosis presented to the clinic with chronic low back pain and left-sided radiculopathy. Her pain significantly increased to excruciating in January 2026, leading to an inability to ambulate. Supportive measures including methylprednisolone, pain regimens including opioids, physical therapy, and aquatic exercise were insufficient in controlling her pain. MRI showed left-sided L2–3 disc herniation, and SPECT-CT demonstrated increased uptake in the entire lumbar spine, but greatest in the L2–3 region. Based on the patient’s imaging and sudden increase in debilitating pain, surgical intervention was indicated. The endoscopic approach was established as the most appropriate option to minimize native tissue damage.

### 3:21 Preoperative Planning and Patient Consent.

The patient’s preoperative T2 MRI was used to create a 3D model of the spine using commercial software segmentation. The L2 and L3 vertebral bodies, L2–3 intervertebral disc, and exiting and traversing nerve roots were segmented to create a 3D image that represented the patient’s unique anatomy. The 3D segmentation allowed objective and specific determination of Kambin’s triangle as defined by the patient’s superior articular process and exiting nerve root. The trajectory was subsequently planned, with a 7.1-mm-diameter space measured in Kambin’s triangle to ensure adequate space for the 6-mm-diameter dilator. Informed consent was obtained from the patient for the endoscopic procedure, as well as for the collection and use of her medical data and surgical video recordings for research and publication purposes. All identifying information was removed to ensure patient anonymity.

### 3:54 Surgical Procedure.

The patient was positioned prone with careful padding of all pressure points. The surgical trajectory was planned using the preoperative segmentation data. The patient was then prepped and draped in a sterile fashion, and bilateral lower extremity EMG electrodes were placed for intraoperative neuromonitoring. The patient was given prophylactic antibiotics. A 2-cm incision was made over the right flank, and two reference pins were placed into the right posterior superior iliac spine (PSIS). An intraoperative CT scan was obtained and merged with the preoperative MRI nerve segmentation using the image fusion tool, where the CT scan was rotated to match the segmentation and the system subsequently aligned the images using an algorithm, with registration confirmed by a technician. The fused images were used to confirm the planned trajectory using the virtual field created with the Brainlab monitoring tools. Using intraoperative navigation and correlation with key anatomical landmarks, a second 2-cm incision was made on the left flank. The neurosurgeon donned the augmented reality glasses, which provided real-time visualization of the target spinal column overlaid on the patient’s back. Using the AR image as a guide, a navigated dilator was advanced toward the L2–3 level along the predetermined trajectory. Confirmation of adequate placement was obtained with AP and lateral x-rays, which confirmed the accuracy of the augmented reality segmentation images, and a hammer was used to advance into the disc space without issue. The navigator was removed and the endoscope was placed for direct visualization. A partial discectomy was performed at the L2–3 level using a rongeur to alleviate pressure on the spinal nerves. After the decompression was performed, the surgeon removed the hardware and a resident reapproximated the surgical incisions bilaterally.

### 5:15 Outcomes and Future Directions.

The use of augmented reality guidance enabled visualization of the segmented nerve roots during the procedure, thus ensuring continuous spatial awareness of the nerve roots during surgery. The 7.1 mm of working space within Kambin’s triangle was provided by preoperative segmentation, a factor that was validated during surgery by the unhindered and smooth advancement of the navigated dilator without the need for foraminoplasty or bony reaming. The integration of both MRI nerve data obtained during preoperative planning and CT imaging during surgery provided a reliable roadmap for surgery. This is especially useful in this patient’s case, in whom a complex rotational deformity resulted from chronic lumbar scoliosis. The patient tolerated the surgery well without any intraoperative complications. There was an immediate improvement in left radiculopathy postoperatively, and the patient was able to walk with significantly reduced pain compared to the preoperative state. There were no new neurological deficits, paresthesias, or neuropathic pain noted in the postoperative period.

The following video was taken 2 hours after surgery [patient follows instructions].

Indications for nerve segmentation paired with augmented reality include patients with severe deformity, previous surgical complications, and those who would benefit from a decreased surgical recovery time. However, endoscopic technology remains expensive, and cost to the patient as well as unique anatomical differences should be taken into consideration. Although endoscopic discectomies increase radiation exposure, use of augmented reality should, in theory, decrease radiation doses by allowing for intraoperative visualization without as many fluoroscopic images needed. Potential complications of this procedure include misalignment of the CT and MRI images, subsequent nerve damage, and longer operative times. Future studies should be performed to validate this technology by comparing AR-guided endoscopic discectomy to traditional techniques and outcomes in larger patient populations. The data collected may be used to create predictive algorithms to determine working corridors and foraminoplasty needs preoperatively. With further refinement of these automated segmentation tools, the requirement for manual segmentation will be reduced and may allow for further dissemination of this technology for use by other surgeons.

## Acknowledgments

Special thanks to Tamra Ames and Matthew Thomas for their assistance with using the Brainlab technology.

## Disclosures

M. Abd-El-Barr reported consulting for Brainlab and Arthrex during the conduct of the study; and consulting for TrackX outside the submitted work.

## Author Contributions

Primary surgeon: Abd-El-Barr. Assistant surgeon: Hatfield. Editing and drafting the video and abstract: Decker, Toussaint, Boukhiam. Critically revising the work: Decker, Toussaint, Hatfield, Abd-El-Barr. Reviewed submitted version of the work: all authors. Approved the final version of the work on behalf of all authors: Decker. Supervision: Decker, Abd-El-Barr.

## Supplemental Information

### Patient Informed Consent

The necessary patient informed consent was obtained in this study.
